# Intrauterine growth retardation affects liver bile acid metabolism in growing pigs: effects associated with the changes of colonic bile acid derivatives

**DOI:** 10.1186/s40104-022-00772-6

**Published:** 2022-11-02

**Authors:** Yang Liu, Md. Abul Kalam Azad, Wanghong Zhang, Liang Xiong, Francois Blachier, Zugong Yu, Xiangfeng Kong

**Affiliations:** 1 Hunan Provincial Key Laboratory of Animal Nutritional Physiology and Metabolic Process, Key Laboratory of Agro-Ecological Processes in Subtropical Region, Institute of Subtropical Agriculture, Chinese Academy of Sciences, Changsha, 410125 Hunan China; 2 College of Veterinary Medicine, Nanjing Agricultural University, Nanjing, 210095 Jiangsu China; 3 UMR PNCA, Université Paris-Saclay, INRAE, 75005 AgroParisTechParis, France

**Keywords:** Bile acid metabolism, Glucolipid metabolism, Intestinal microbiota, Intrauterine growth retardation, Pigs

## Abstract

**Background:**

Intrauterine growth retardation (IUGR) is associated with severely impaired nutrient metabolism and intestinal development of pigs. Our previous study found that IUGR altered intestinal microbiota and metabolites in the colon. However, the consequences of IUGR on bile acid metabolism in pigs remained unclear. The present study aimed to investigate the bile acid metabolism in the liver and the profile of bile acid derivatives in the colon of growing pigs with IUGR using bile acid targeted metabolomics. Furthermore, we determined correlations between colonic microbiota composition and metabolites of IUGR and normal birth weight (NBW) pigs at different growth stages that were 7, 21, and 28-day-old, and the average body weight (BW) of 25, 50, and 100 kg of the NBW pigs.

**Results:**

The results showed that the plasma total bile acid concentration was higher (*P* < 0.05) at the 25 kg BW stage and tended to increase (*P* = 0.08) at 28-day-old in IUGR pigs. The hepatic gene expressions related to bile acid synthesis (*CYP7A1*, *CYP27A1*, and *NTCP*) were up-regulated (*P* < 0.05), and the genes related to glucose and lipid metabolism (*ATGL*, *HSL*, and *PC*) were down-regulated (*P* < 0.05) at the 25 kg BW stage in IUGR pigs when compared with the NBW group. Targeted metabolomics analysis showed that 29 bile acids and related compounds were detected in the colon of pigs. The colonic concentrations of dehydrolithocholic acid and apocholic acid were increased (*P* < 0.05), while isodeoxycholic acid and 6,7-diketolithocholic acid were decreased (*P* < 0.05) in IUGR pigs, when compared with the NBW pigs at the 25 kg BW stage. Moreover, Spearman’s correlation analysis revealed that colonic *Unclassified*_[*Mogibacteriaceae*], *Lachnospira,* and *Slackia* abundances were negatively correlated (*P* < 0.05) with dehydrolithocholic acid, as well as the *Unclassified*_*Clostridiaceae* abundance with 6,7-diketolithocholic acid at the 25 kg BW stage.

**Conclusions:**

These findings suggest that IUGR could affect bile acid and glucolipid metabolism in growing pigs, especially at the 25 kg BW stage, these effects being paralleled by a modification of bile acid derivatives concentrations in the colonic content. The plausible links between these modified parameters are discussed.

**Supplementary Information:**

The online version contains supplementary material available at 10.1186/s40104-022-00772-6.

## Background

Pigs are susceptible to intrauterine growth retardation (IUGR) among mammals since it represents 15%–20% of the total birth of newborn piglets [1]. The IUGR is known to reduce the postnatal growth performance and efficiency of nutrient utilization later in life [2]. Several studies also confirmed that IUGR reduced the intestine weight at birth and damaged its morphological structure, function, and microbiota colonization [3]. Moreover, the IUGR can alter colonic microbial diversity and fermentation activity and associated biochemical processes [4]. In addition, some bacterial species of the intestinal microbiota of IUGR pig, such as *Lactobacillus*, showed a negative correlation with the growth performance, while *Unclassified*_*Ruminococcaceae* was positively correlated [5]. Colon harbors the largest concentration of bacteria in mammals, including pigs, and these bacteria produce a variety of metabolites, which play an important role in the host physiology and pathophysiology, notably in the large intestine [6], but also in the development of metabolic disease [7]. Recent studies showed that IUGR altered several bacterial metabolites with known biological activities, including short-chain fatty acids, bile acid derivatives, and indole derivatives [8]. Therefore, IUGR affects intestinal microbiota and its metabolic activity in pigs; however, further studies aiming at the impacts of IUGR on both the host and microbiota metabolism are necessary to improve the growth and health of IUGR pigs and reduce economic loss.

Bile acids have recently become an emerging research hot spot due to their potential roles as metabolic regulators and molecular signals regulating whole-body metabolic homeostasis. These compounds have been widely used in animal production as prominent feed additives [9]. Bile acids have also been reported to inhibit bacterial growth [10]. The bacteriostatic effects of the deoxycholic acid derived from the bacterial transformation of bile acid were tenfold higher than those of the primary bile acids, indicating that the profile of bile acids and their derivatives can exert survival pressure on some particular microbes [11]. Several intestinal bacteria, such as *Bacteroides*, *Clostridium*, *Lactobacillus*, *Bifidobacterium*, and *Listeria*, are closely related to bile acid metabolisms [12]. Intestinal microbiota promotes the deconjugation, dehydrogenation, and dehydroxylation of primary bile acids to generate a variety of secondary bile acids [13], which are one of the active metabolites in the colon [14]. Compared with the germ-free piglets, the fecal transplanted piglets displayed an increased secretion of bile acids and derivatives, resulting in different bile acid profiles [15]. Dietary supplementation with bile acid-binding resin increased the abundance of Firmicutes and decreased that of *Bacteroides* [16]. Considering the abnormal metabolism of IUGR pigs, it appears timely to investigate the roles of bile acids and their receptors in regulating metabolisms of IUGR pigs.

Our previous studies showed that IUGR altered the intestinal microbial community and their metabolites at 7, 21, and 28-day-old [5] and 25, 50, and 100 kg body weight (BW) stages [17], but the effect of these changes on bile acid metabolism in IUGR pigs remained unknown. Therefore, we hypothesized that bile acid metabolism in IUGR pigs might be different from normal birth weight (NBW) pigs. Thus, we compared the concentrations of total bile acids in plasma and bile acid derivatives in colon contents, and the expressions of genes related to bile acid synthesis and bile acid receptors, as well as glucolipid metabolism in IUGR pigs and NBW pigs at different growth stages.

## Materials and methods

### Experimental design, animals, and diets

All pigs treated in this study were from our previous studies [5, 17]. All sows (Large White × Landrace) were selected from an experimental herd located in Yong’an Town, Liuyang City, Hunan Province, China. Two independent experiments were conducted in the present study, and the sows were from two independent cohorts of sows. The sows were fed pregnant sows’ diet during 1−85 days of pregnancy, and the lactating sows’ diet was supplied from day 86 of pregnancy to day 21 after delivery (Additional file 1: Table S1). Piglets with a birth weight higher than the mean birth weight of each litter were classified as NBW piglets, and piglets within the 10% lower than the mean birth weight of each litter were classified as IUGR piglets [5]. In the neonatal phase, a total of 48 piglets were obtained from 24 litters, including one IUGR piglet and one NBW piglet per litter [17]. All suckling piglets were kept in a warm thermal container and fed by sows freely. The commercial creep feed was supplied during 5–21-day-old (Additional file 2: Table S2). Piglets were weaned at 21-day-old and transferred into the nursery pens. The piglets had 24 h access to nursery pig feed and water. At 7, 21, and 28-day-old after birth, 16 piglets (eight pairs of one NBW piglet and one IUGR littermate) were weighed after 12 h of fasting and then euthanized for sample collection. In the growing-finishing phase, a total of 72 castrated male piglets were obtained from 36 litters, including one IUGR and one NBW piglet per litter [5]. All piglets were weaned at 21-day-old and transferred to the nursery facility pens with free access to commercial weaning diets and water. A nursery diet was fed during 28–69-day-old, a growing diet during 70–103-day-old, and a finishing diet during 104–165-day-old (Additional file 3: Table S3). This trial was completed when the average body weight of NBW pigs reached at 25, 50, and 100 kg, respectively. The pigs were individually penned (0.6 m × 1.2 m) with stainless steel pens over a totally slotted floor to reduce the influence of mutual attack and psychological stress of weaned piglets. Each pen was equipped with a single-hole feeder and a water nipple, and no antibiotics were used during the entire study. The composition and nutrient levels of diets for sows and piglets met the NRC (2012) recommended requirements [18].

### Sample collection

The samples were obtained for analyzing microbiota, bile acids, and gene expression in this study in accordance with our previous studies [5, 17]. Sixteen piglets (eight pairs; one NBW and one IUGR littermate) from the early stage were weighed and euthanized (at 7, 21, and 28-day-old, respectively) by exsanguination after general anesthesia (intravenous injection of 4% sodium pentobarbital solution, 40 mg/kg BW) for sample collection after 2 h from the last suckling. When the average BW of NBW pigs reached at 25, 50, and 100 kg in the growing-finishing stage, 24 pigs (12 NBW and 12 IUGR) per BW stage were randomly weighed and euthanized using electrical stunning (120 V, 200 Hz) after 12 h of fasting. Blood samples (5 mL) were collected from the precaval vein into heparin-treated tubes, and the plasma was obtained by centrifuging at 3500 × *g* for 10 min at 4 °C, and then immediately stored at − 20 °C for plasma total bile acid analysis. The luminal contents of the colon (middle position) were collected, immediately frozen in liquid nitrogen, and then stored at − 80 °C for targeted metabolomics analysis. In addition, the colonic tissues were excised and rinsed with ice-cold physiological saline, liver and intestinal mucosa samples were immediately frozen in liquid nitrogen, and then stored at − 80 °C for total RNA extraction.

### Determination of plasma total bile acid concentrations

The plasma samples were thawed and centrifuged at 3500 × *g* for 10 min at 4 °C. The supernatants were used to assay total bile acids concentrations using the Total Bile Acids Assay Kit (MedicaSystem, Ningbo, China) by a Fuautomatic Biochemical Analyzer (Beckman, USA).

### Metabolites extraction and UHPLC-PRM-MS analysis

Samples obtained from the IUGR and NBW pigs were precisely weighed (25 mg) and mixed with 1000 μL of extract solution (precooled at 4 °C, acetonitrile-methanol–water, 2:2:1, containing 0.1% formic acid and isotopically-labeled internal standard mixture). The samples were homogenized at 35 Hz for 4 min and sonicated for 5 min in an ice-water bath. The homogenization and sonication cycles were repeated three times, followed by centrifugation at 12,000 × *g* for 15 min. The final supernatants were proceeded using an ultra-high performance liquid chromatography (UHPLC) System (Vanquish, Thermo Fisher Scientific, MA, USA), equipped with a Waters ACQUITY UPLC BEH C18 column (150 mm × 2.1 mm, 1.7 μm). The mobile phase A contained 1 mmol/L ammonium acetate and 0.1% acetic acid in water, and the mobile phase B contained acetonitrile. The column temperature was set at 50 °C. The auto-sampler temperature was set at 4 °C, and the injection volume was 1 μL.

A Q Exactive HFX mass spectrometer (Thermo Fisher Scientific, MA, USA) was applied for assay development. Typical ion source parameters were: spray voltage =  + 3500/ − 3100 V, sheath gas (N_2_) flow rate = 40, aux gas (N_2_) flow rate = 15, sweep gas (N_2_) flow rate = 0, aux gas (N_2_) temperature = 350 °C, and capillary temperature = 320 °C. The parallel reaction monitoring (PRM) parameters for each of the targeted analytes were optimized by injecting the standard solutions of the individual analytes into the API source of the mass spectrometer. The results of extracted ion chromatographs (EICs) from a standard solution and a sample showed that most analytes showed excellent peak shapes, and good separations were obtained. The lower limits of detection (LLODs) ranged from 0.24 to 15.62 nmol/L, and the lower limits of quantitation (LLOQs) ranged from 0.49 to 31.25 nmol/L for all the analytes. Correlation coefficients (R^2^) of regression fitting were above 0.98 for all the analytes. The analytical recoveries and relative standard deviations of the QC samples were with six technical replicates. The recoveries determined were 80.00%–113.30% for all the analytes, with all the relative standard deviation (RSDs) below 18.70%. The analysis metrics indicated that the method allowed accurate quantitation of the targeted metabolites in the colonic contents in the concentration range described above.

### Targeted metabolomics data analysis

The positive and negative ion modes from the mass spectrometer were performed for all samples. A total of 6405 peaks were extracted from all samples, and 3937 peaks were retained after preprocessing. The resulting peaks were imported to SIMCA software (V15.0.2, Sartorius Stedim Data Analytics AB, Umea, Sweden). The principal component analysis (PCA) was carried out to visualize the distribution and the grouping of the samples. A 95% confidence interval in the PCA score plot was used as the threshold to identify potential outliers in the dataset. The orthogonal projections to latent structures-discriminate analysis (OPLS-DA) was applied to visualize group separation and find significant metabolites, and sevenfold permutation tests in the OPLS-DA model were used to verify model validity and robustness. The metabolites with VIP > 1 and *P* < 0.05 (student’s *t*-test) were considered as significantly changed metabolites.

### Correlation analysis between colonic bile acid metabolism and microbiota

The results of 16S rRNA sequencing of the intestinal microbiota (unpublished data) were from our research lab. The colonic microbial DNA was extracted using a HiPure Stool DNA Kit (Magen, Guangzhou, China), following the manufacturer’s instructions. The final DNA concentration and purification were determined using a NanoDrop 1000 UV–vis spectrophotometer (Thermo Fisher Scientific, Waltham, MA, USA), and DNA quality was checked by 1% agarose gel electrophoresis. The V3–V4 hypervariable regions of the bacterial 16S rRNA gene were amplified with the primers 338F (5′-ACTCCTACGGGAGGCAGCAG-3′) and 806R (5′-GGACTACHVGGGTWTCTAAT-3′) using a thermocycler PCR system (GeneAmp 9700; Thermo Fisher Scientific). The resultant PCR products were extracted using a 2% agarose gel and further purified using the AxyPrep DNA Gel Extraction Kit (Axygen Biosciences, Union City, CA, USA) and quantified using QuantiFluorTM-ST (Promega, Madison, WI, USA) according to the manufacturer’s protocol. Purified amplicons were pooled in equimolar amounts, paired-end sequenced (2 × 300 bp) on an Illumina MiSeq platform (Illumina, San Diego, CA, USA), and analyzed by Majorbio Bio-pharm Technology Co., Ltd (Shanghai, China). The linear discriminant analysis effect size (LEfSe) was used to compare differences at the genus level between IUGR pigs and NBW pigs at different growth stages. Spearman’s correlation was evaluated between colonic microbiota and bile acid derivatives.

### Total RNA extraction and qPCR analysis

The total RNA was isolated from liver and colonic mucosa samples using reagent TRIzol (Invitrogen, Shanghai, China) according to manufacturers’ instructions. The total RNA (500 ng) was used as a template for the cDNA reaction, which was synthesized using the AG reagent kit with gDNA Eraser (AG, Changsha, China). The real-time quantitative PCR (RT-qPCR) was used to detect the gene expression and performed in the Light-Cycler® 480 II Real-Time PCR System (Roche, Basel, Switzerland). Pig-specific primers were designed and synthesized by the Sangon Biotech Co., Ltd (Shanghai, China) (Table 1). The reaction mixture (10 μL) consisted of 5.0 μL SYBR Premix Ex TaqTM (AG, Changsha, China), 2.0 μL of template cDNA, 0.25 μL of each primer, and 2.5 μL of RNase free ddH_2_O. The RT-qPCR reaction conditions were as follows: denaturation at 95 °C for 5 min; 40 cycles of denaturation at 95 °C for 5 s and annealing at 60 °C for 30 s; and dissociation stage. The relative abundance of the gene expression was analyzed using the 2^−ΔΔCt^ method, and the housekeeping gene β-actin was used for internal control [15].

**Table 1:** Pig-specific primers used to detect colonic mucosa and liver-related genes

Gene name	Primers (5′ to 3′)	Amplicon size, bp	Accession No.
*ABST*	F: AAGTTCCTGGGGCACGTAAA	101	NM_000452.3
	R: CTCCTGGACAGCATCCCATT		
*ATGL*	F: ACCTGTCCAACCTGCTGC	163	NM_001098605.1
	R: GCCTGTCTGCTCCTTTATCCA		
*BESP*	F: CGCAGCGTGAAGAAATGTGG	126	XM_003133457.5
	R: AAAACCGAAACAGTTGAAAGAGGC		
*CYP7A1*	F: GTGTTCAAGACGGGCCACTA	120	NM_001005352.3
	R: GGAGCGACTTGGCTTTCTCT		
*CYP8B1*	F: GCAGGCGGAGGAGTTATTCA	130	NM_214426.1
	R: TTATGCCGTGCCTCTCCAAG		
*CYP27A1*	F: TTGAGAAACGCATTGGCTGC	90	NM_001243304.1
	R: ATCCAGGTATCGCCTCCAGT		
*FAS*	F: ATAAGTTCGACGTCAGCCCG	88	NM_001099930.1
	R: TAGCCAGTGCCAGGGAAAAG		
*FXR*	F: TATGAACTCAGGCGAATGCCTGCT	150	NM_001287412.1
	R: ATCCAGATGCTCTGTCTCCGCAAA		
*FGF19*	F: TGAGTACCGTGGCGATCAAG	108	XM_003122420.3
	R: GCGGATCTCCTCCTCGAAAG		
*G6PC*	F: AAGCCAAGCGAAGGTGTGAGC	158	NM_001113445.1
	R: AAGCATTCAGCCAACA		
*HSL*	F: ACCCTCGGCTGTCAACTTCTT	152	NM_214315.3
	R: TCCTCCTTGGTGCTAATCTCGT		
*IBABP*	F: GCAAGGAGTGCGACATAGAGAC	102	NM_001040442.1
	R: TGGTGGTAGTTGGGGCTGTT		
*MRP2*	F: GAACAGGTTTGCTGGCGATATT	121	XM_021073710.1
	R: GCCAGGAGCGCAAAGACA		
*NTCP*	F: TGTCATCAAGGGAGGAACGA	139	XM_001927695.5
	R: CGAGCATTGAGGCGGAAAAG		
*PCK2*	F: ACAGGAGGTTCGTGACATTCGG	172	NM_001161753.1
	R: GTGGTGCTGTGCTCACTTGCTA		
*PC*	F: CCGCAAGATGGGAGACAAGGT	164	NM_214349.1
	R: GGAAGCCGTAGGTGTTGGAGAA		
*TGR5*	F: TGATCATCGCTACACAGCCC	124	NM_001077191.2
	R: TCAAGTCAAGGTCCACGCTG		

*ABST,* Apical sodium dependent bile acid transporter; *ATGL,* Adipose triglyceride lipase; *BESP,* Bile salt export pump; *CYP7A1,* Cytochrome P450 family 7 subfamily A member 1; *CYP8B1,* Cytochrome P450 family 8 subfamily B member 1; *FAS,* Fatty acid synthetase; *FGF19,* Fibroblast growth factor 19; *FXR,* Farnesoid x receptor; *G6PC,* Glucose-6-Phosphatase; *HSL,* Hormone-sensitive lipase; *IBABP,* Ileal bile acid binding protein; *MRP2,*  Multidrug resistance protein 2; *NTCP,* Sodium taurocholate cotransporting polypeptide; *PC,* Pyruvate carboxylase; *PCK2,* Phosphoenolpyruvate carboxykinase 2; *TGR5,* G protein-coupled bile acid receptor 1

### Statistical analysis

All data except microbial data were analyzed using SPSS 22.0 statistical software (SPSS Inc., Chicago, IL, USA). Data were analyzed by one-way ANOVA, and comparative analyses were conducted using the Tukey post-hoc test. Colonic bile acid derivatives were assessed by the Student’s *t*-test. Statistical results are presented as means ± standard error of the mean (SEM). Differences were considered significant if *P* < 0.05 and trends when 0.05 ≤ *P* < 0.10. GraphPad Prism 8.00 (San Diego, CA, USA) was used to make bar charts. Spearman’s correlation was evaluated using the R package ggplot2 3.3.1 (https://www.r-project.org/).

## Results

### Effects of IUGR on plasma total bile acids concentrations at different growth stages

**Fig. 1:**
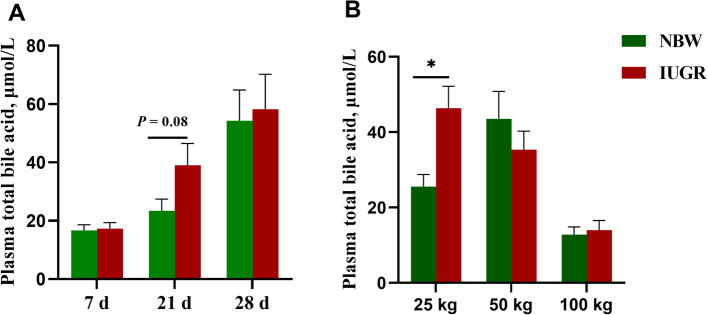
Plasma total bile acid concentration of intrauterine growth retardation (IUGR) pigs and normal birth weight (NBW) pigs at different growth stages. Data are expressed as means ± SEM (*n* = 8−12). *P < 0.05. 7 d, 21 d, and 28 d represent 7, 21, and 28-day-old, respectively; 25 kg, 50 kg, and 100 kg represent the average BW of NBW pigs reached at 25, 50, and 100 kg BW, respectively

The effects of IUGR on plasma total bile acids concentrations are presented in Fig. 1. The IUGR pigs displayed a higher (*P* < 0.05) plasma concentration of total bile acids at the 25 kg BW stage and a trend for an increased (*P* = 0.08) plasma total bile acids at 21-day-old, when compared with the NBW pigs.

### Effects of IUGR on the composition of colonic bile acid derivatives

**Fig. 2:**
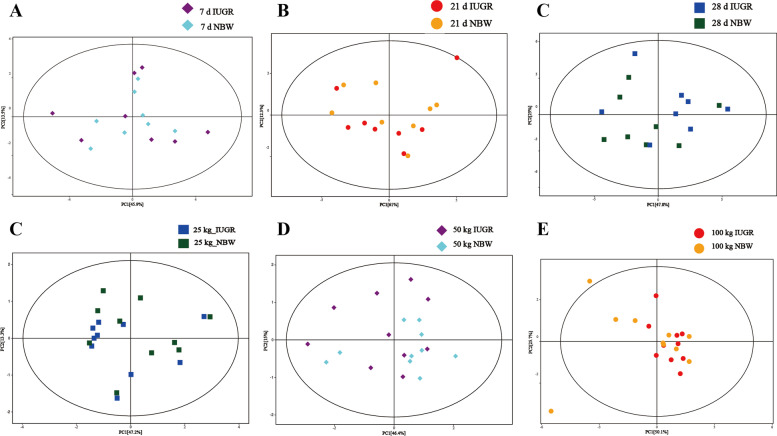
The PCA score plots of colonic contents targeted metabolomics data obtained by LC–MS for intrauterine growth retardation (IUGR) pigs and normal birth weight (NBW) pigs at different growth stages (*n* = 8−10). (**A)**, (**B)**, and (**C**) represent 7, 21, and 28-day-old, respectively; (**D)**, (**E**), and (**F)** represent the average BW of NBW pigs reached at 25, 50, and 100 kg BW, respectively

**Fig. 3:**
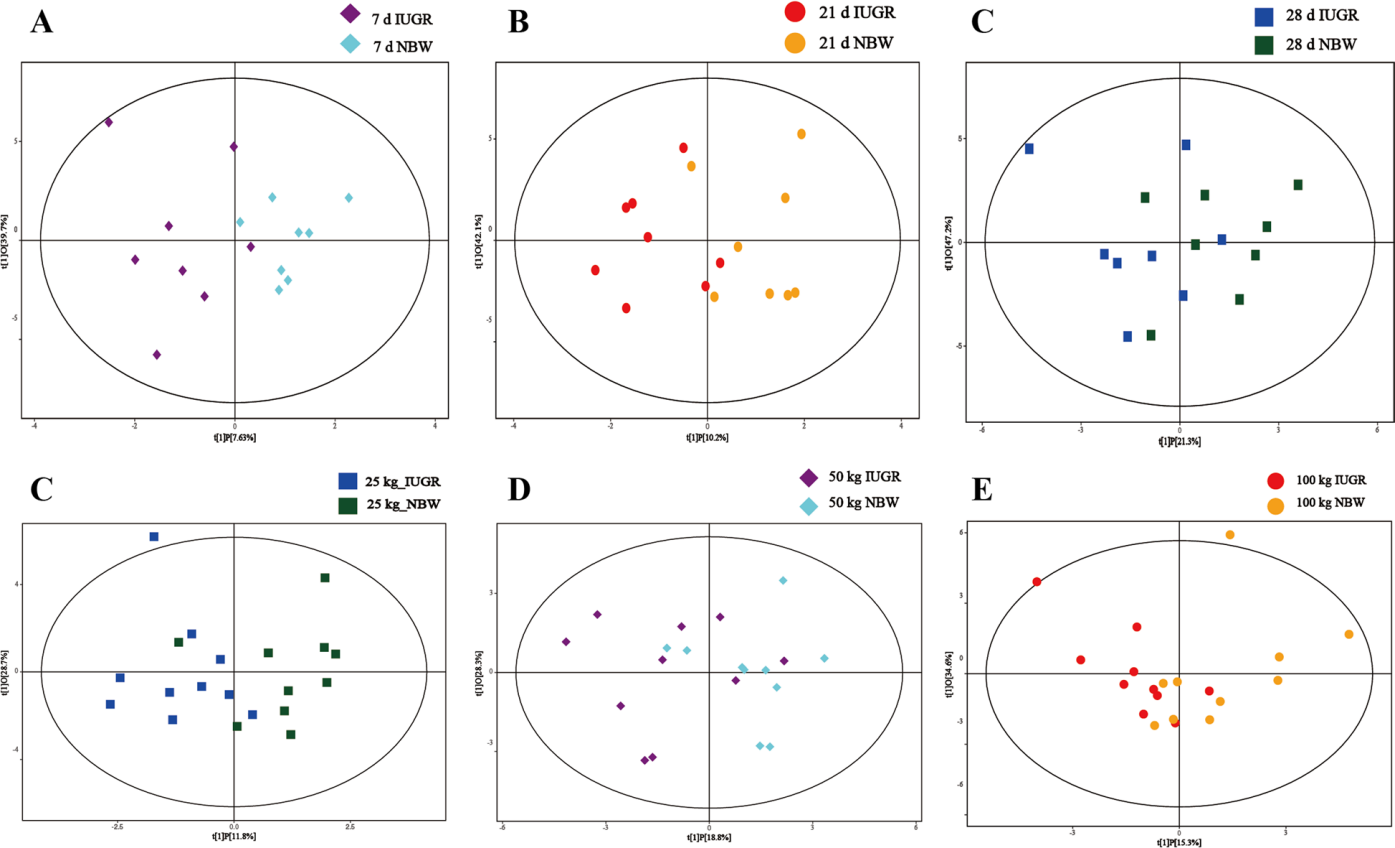
The OPLS-DA score plots of colonic contents targeted metabolomics data obtained by LC–MS for intrauterine growth retardation (IUGR) pigs and normal birth weight (NBW) pigs at different growth stages (*n* = 8−10). R^2^Y = 0.99; Q^2^ = 0.95. (**A)**, (**B**), and (**C**) represent 7, 21, and 28-day-old, respectively; (**D**), (**E**), and (**F**) represent the average BW of NBW pigs reached at 25, 50, and 100 kg BW, respectively

**Fig. 4:**
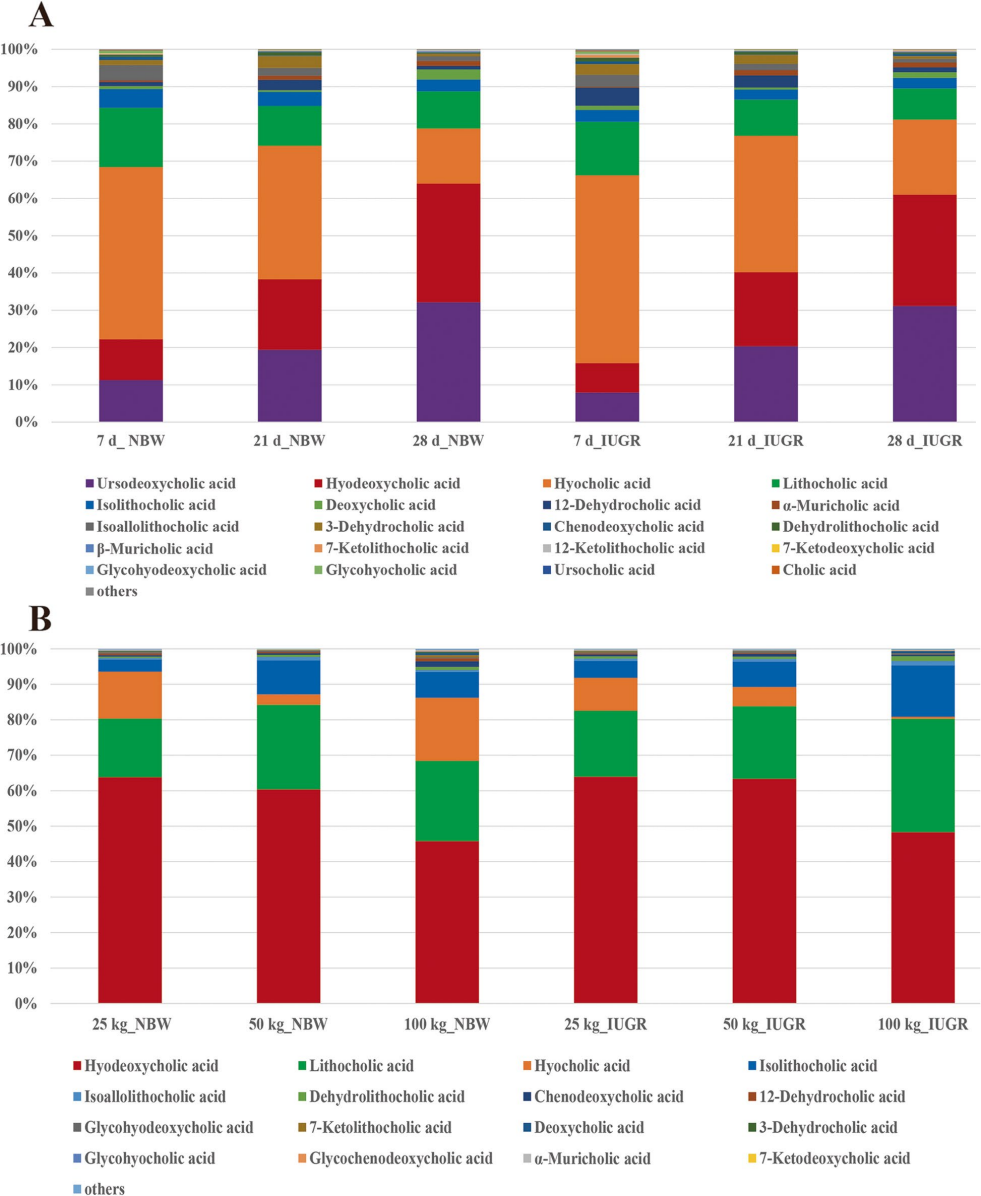
Colonic bile acid composition of intrauterine growth retardation (IUGR) pigs and normal birth weight (NBW) pigs at different growth stages (*n* = 8−10). (**A)** Compositions of bile acid derivatives in colon at 7, 21, and 28-day-old. (**B)** Compositions of bile acid derivatives in colon at the 25, 50, and 100 kg BW stages. 7 d, 21 d, and 28 d represent 7, 21, and 28-day-old, respectively; 25 kg, 50 kg, and 100 kg represent the stages when the average BW of NBW pigs reached at 25, 50, and 100 kg BW, respectively

The PCA results revealed that there were no clear separations between the NBW pigs and IUGR pigs at different growth stages (Fig. 2). The OPLS-DA score plots (based on unweighted UniFrac distances) showed a tendency to cluster into two groups between the IUGR pigs and NBW pigs (Fig. 3). The results of target analysis for colonic bile acids are presented in Fig. 4. A total of 29 bile acids were detected using the targeted bile acid metabolomics analysis (VIP > 1.0 and adjusted *P* < 0.10). At 7-day-old, hyocholic acid (45%), lithocholic acid (13%), ursodeoxycholic acid (11%), hyodeoxycholic acid (10%), and isolithocholic acid (3%) were the most abundant metabolites in the colonic contents of the NBW pigs, and hyocholic acid (50%), lithocholic acid (14%), ursodeoxycholic acid (9%), hyodeoxycholic acid (8%), 12-dehydrocholic acid (5%), and isolithocholic acid (2%) were the most abundant metabolites in the IUGR pigs. At 21-day-old, hyocholic acid (32%), ursodeoxycholic acid (19%), hyodeoxycholic acid (18%), lithocholic acid (8%), and isolithocholic acid (2%) were the most abundant metabolites in the colonic contents of the NBW pigs, and hyocholic acid (38%), ursodeoxycholic acid (21%), hyodeoxycholic acid (19%), lithocholic acid (10%), 12-dehydrocholic acid (4%), and isolithocholic acid (3%) were the most abundant metabolites in the IUGR pigs. At 28-day-old, ursodeoxycholic acid (32%), hyodeoxycholic acid (30%), hyocholic acid (15%), and lithocholic acid (11%) were the most abundant metabolites in the NBW pigs, and ursodeoxycholic acid (31%), hyodeoxycholic acid (30%), hyocholic acid (20%), lithocholic acid (9%), and isolithocholic acid (2%) were the most abundant metabolites in the IUGR pigs (Fig. 4A).

At the 25 kg BW stage (Fig. 4B), hyodeoxycholic acid (63%), lithocholic acid (17%), hyocholic acid (14%), and isolithocholic acid (2%) were the most abundant metabolites in the NBW pigs, and hyodeoxycholic acid (64%), lithocholic acid (19%), hyocholic acid (10%), and isolithocholic acid (6%) were the most abundant metabolites in the IUGR pigs. At the 50 kg BW stage, hyodeoxycholic acid (60%), lithocholic acid (25%), isolithocholic acid (11%), and hyocholic acid (2%) were the most abundant metabolites in the NBW pigs, and hyodeoxycholic acid (63%), lithocholic acid (21%), isolithocholic acid (7%), and hyocholic acid (6%) were the most abundant metabolites in the IUGR pigs. At the 100 kg BW stage, hyodeoxycholic acid (45%), lithocholic acid (22%), hyocholic acid (17%), isolithocholic acid (9%), and chenodeoxycholic acid (2%) were the most abundant metabolites in the NBW pigs, and hyodeoxycholic acid (48%), lithocholic acid (32%), isolithocholic acid (15%), and dehydrolithocholic acid (2%) were the most abundant metabolites in the IUGR pigs.

### Different bile acid derivatives in colon contents of IUGR pigs and NBW pigs

**Fig. 5:**
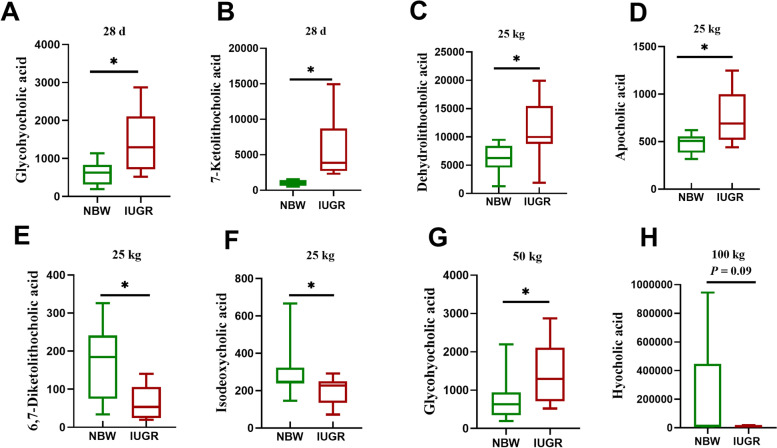
Differences of bile acid derivatives in the colonic contents of intrauterine growth retardation (IUGR) pigs and normal birth weight (NBW) pigs at different growth stages. Data are presented as means ± SEM (*n* = 8−10). *P < 0.05. (**A)** and (**B**) represent the concentrations of bile acids in NBW and IUGR pigs at 28-day-old; (**C**), (**D**), (**E**), and (**F**) represent the concentrations of bile acids in NBW and IUGR pigs, when the average BW of NBW pigs reached at 25 kg; (**G**), represents the concentrations of bile acids in NBW and IUGR pigs, when the average BW of NBW pigs reached at 50 kg; (**H**), represents the concentrations of bile acids in NBW and IUGR pigs, when the average BW of NBW pigs reached at 100 kg

The differences of bile acid derivatives in NBW pigs and IUGR pigs are shown in Fig. 5. Compared with the NBW group, the colonic concentrations of glycohyocholic acid and 7-ketolithocholic acid in the IUGR pigs were increased (*P* < 0.05) at 28-day-old, and glycohyocholic acid was increased (*P* < 0.05) at the 50 kg BW stage. At the 25 kg BW stage, the IUGR pigs had higher (*P* < 0.05) colonic concentrations of dehydrolithocholic acid and apocholic acid and lower (*P* < 0.05) isodeoxycholic acid and 6,7-diketolithocholic acid compared with the NBW pigs. At the 100 kg BW stage, the colonic concentration of hyocholic acid tended to decrease (*P* = 0.09) in the IUGR pigs compared with the NBW pigs.

### Effects of different growth stages on colonic bile acid derivatives in IUGR pigs and NBW pigs

The differences of colonic bile acid derivatives in NBW and IUGR pigs at different growth stages are shown in Table 2 and 3. At the suckling-weaning stage, in the NBW pigs, the concentrations of dehydrolithocholic acid, glycohyodeoxycholic acid, and lithocholic acid were increased (*P* < 0.05), and the hyodeoxycholic acid (*P* = 0.06), isolithocholic acid (*P* = 0.09), ursodeoxycholic acid (*P* = 0.06), and β-muricholic acid (*P* = 0.06) were tended to increase at 21-day-old compared with those at 7-day-old; the concentrations of glycohyodeoxycholic acid, hyodeoxycholic acid, lithocholic acid, ursodeoxycholic acid, and ursocholic acid were increased (*P* < 0.05), while glycochenodeoxycholic acid was tended to decrease (*P* = 0.07) at 28-day-old compared with those at 7-day-old; the colonic concentrations of hyodeoxycholic acid (*P* = 0.09), taurohyodeoxycholic acid (*P* = 0.09), and ursocholic acid (*P* < 0.05) were increased at 28-day-old compared with those at 21-day-old. In the IUGR pigs, the concentrations of glycohyodeoxycholic acid and taurohyodeoxycholic acid were increased (*P* < 0.05), and chenodeoxycholic acid (*P* = 0.08), hyodeoxycholic acid (*P* = 0.08), isolithocholic acid (*P* = 0.08), ursodeoxycholic acid (*P* = 0.07), and β-muricholic acid (*P* = 0.06) had an increasing trend, while the concentrations of glycochenodeoxycholic acid (*P* < 0.05) and glycohyocholic acid (*P* = 0.06) were decreased at 21-day-old compared with those at 7-day-old; the concentrations of glycohyodeoxycholic acid, glycodeoxycholic acid, hyodeoxycholic acid, lithocholic acid, taurohyodeoxycholic acid, and ursodeoxycholic acid were increased (*P* < 0.05) and ursocholic acid (*P* = 0.06), and β-muricholic acid (*P* = 0.06) had an increasing trend, while glycochenodeoxycholic acid was decreased (*P* < 0.05) at 28-day-old compared with those at 7-day-old; the concentrations of cholic acid, hyodeoxycholic acid, taurohyodeoxycholic acid, and ursodeoxycholic acid were increased (*P* < 0.05), and glycohyodeoxycholic acid (*P* = 0.09) and ursocholic acid (*P* = 0.07) displayed an increasing trend at 28-day-old compared with those at 21-day-old.

**Table 2:** Effect of intrauterine growth retardation (IUGR) on bile acid derivatives in colonic contents of pigs at piglet stages

Item, nmol/g	Groups	7 d	21 d	28 d
Apocholic acid	NBW	0.04 ± 0.03	0.09 ± 0.03	0.17 ± 0.26
	IUGR	0.42 ± 0.29	0.07 ± 0.05	0.12 ± 0.09
Cholic acid	NBW	0.37 ± 0.18	0.43 ± 0.11	0.68 ± 0.23
	IUGR	0.58 ± 0.35^b^	0.30 ± 0.05^b^	1.28 ± 0.38^a^
Dehydrolithocholic acid	NBW	1.91 ± 0.82^b^	12.65 ± 3.91^a^	6.97 ± 2.72^ab^
	IUGR	4.28 ± 1.84	8.14 ± 1.86	10.73 ± 6.27
Glycochenodeoxycholic acid	NBW	0.63 ± 0.09	0.31 ± 0.11	0.27 ± 0.09
	IUGR	0.76 ± 0.21	0.23 ± 0.08	0.26 ± 0.09
Glycohyodeoxycholic acid	NBW	0.17 ± 0.03	0.63 ± 0.09	1.39 ± 0.45
	IUGR	0.19 ± 0.08	0.45 ± 0.08	1.60 ± 0.57
Hyodeoxycholic acid	NBW	37.76 ± 10.55^b^	247.54 ± 94.80^ab^	779.47 ± 270.20^a^
	IUGR	28.90 ± 10.42^b^	216.70 ± 91.79^b^	906.24 ± 267.16^a^
Hyocholic acid	NBW	159.60 ± 87.02	116.88 ± 8.78	50.83 ± 40.58
	IUGR	184.73 ± 80.98	119.27 ± 61.12	315.06 ± 128.99
Isoallolithocholic acid	NBW	14.09 ± 5.78^b^	27.35 ± 4.93^ab^	30.02 ± 9.56^a^
	IUGR	11.79 ± 2.7	18.30 ± 9.53	28.52 ± 11.11
Isolithocholic acid	NBW	17.45 ± 7.89^b^	49.41 ± 15.69^ab^	76.39 ± 29.96^a^
	IUGR	11.45 ± 4.74	29.45 ± 8.32	84.17 ± 42.17
Lithocholic acid	NBW	54.92 ± 17.72^b^	139.63 ± 35.88^ab^	245.69 ± 72.42^a^
	IUGR	52.82 ± 19.30^b^	106.29 ± 31.44^ab^	254.22 ± 79.72^a^
α-muricholic acid	NBW	1.56 ± 0.41	13.6 ± 9.93	34.06 ± 24.64
	IUGR	0.94 ± 0.33^b^	15.62 ± 10.26^ab^	39.78 ± 22.77^a^
β-muricholic acid	NBW	0.63 ± 0.11^b^	3.69 ± 1.59^b^	10.09 ± 4.96^a^
	IUGR	0.30 ± 0.03^b^	2.67 ± 1.14^b^	10.55 ± 4.39^a^
Ursocholic acid	NBW	0.19 ± 0.06^b^	0.37 ± 0.16^b^	0.92 ± 0.23^a^
	IUGR	0.21 ± 0.11^b^	0.22 ± 0.07^b^	1.30 ± 0.49^a^
Ursodeoxycholic acid	NBW	39.12 ± 10.44^b^	254.21 ± 97.32^b^	790.07 ± 275.45^a^
	IUGR	29.29 ± 10.42^b^	222.67 ± 93.55^b^	944.53 ± 279.74^a^

Data are presented as means ± SEM (*n* = 8–10). ^a−b^Different superscript letters in the same row showed significantly different at 7, 21, and 28-day-old (*P* < 0.05). 7, 21, and 28 d represent 7, 21, and 28-day-old, respectively. *NBW*, normal birth weight; *IUGR*, intrauterine growth retardation

**Table 3:** Effect of intrauterine growth retardation (IUGR) on bile acid derivatives in colonic contents of pigs at growing-finishing stages

Item, nmol/g	Groups	25 kg	50 kg	100 kg
Apocholic acid	NBW	0.59 ± 0.13^ab^	0.41 ± 0.10^b^	1.04 ± 0.29^a^
	IUGR	1.05 ± 0.29	0.36 ± 0.09	1.06 ± 0.50
Cholic acid	NBW	0.38 ± 0.12	0.24 ± 0.02	0.50 ± 0.15
	IUGR	0.36 ± 0.11^b^	0.30 ± 0.05^b^	0.28 ± 0.03^a^
Dehydrolithocholic acid	NBW	7.21 ± 1.40^b^	8.07 ± 1.19^b^	16.49 ± 4.07^a^
	IUGR	11.06 ± 1.56^b^	8.87 ± 2.69^b^	21.08 ± 4.45^a^
Glycochenodeoxycholic acid	NBW	1.26 ± 0.18^b^	0.68 ± 0.09^b^	3.56 ± 1.68^a^
	IUGR	0.76 ± 0.21	0.23 ± 0.08	0.26 ± 0.09
Glycohyodeoxycholic acid	NBW	6.34 ± 0.75^a^	3.23 ± 0.51^b^	5.58 ± 2.21^ab^
	IUGR	0.19 ± 0.08	0.45 ± 0.08	1.60 ± 0.57
Hyodeoxycholic acid	NBW	1198.85 ± 200.75	856.68 ± 148.08	896.71 ± 217.13
	IUGR	1152.44 ± 199.17	900.32 ± 158.43	61.85 ± 207.73
Hyocholic acid	NBW	23.06 ± 8.01	6.20 ± 1.50	13.2 ± 6.41
	IUGR	9.68 ± 2.22	20.92 ± 7.95	8.47 ± 1.69
Isoallolithocholic acid	NBW	8.07 ± 2.26	15.37 ± 2.63	10.98 ± 1.86
	IUGR	12.97 ± 4.72	10.72 ± 2.19	19.14 ± 4.54
Isolithocholic acid	NBW	63.54 ± 11.67^b^	134.43 ± 15.11^a^	143.25 ± 21.82^a^
	IUGR	83.70 ± 14.70^b^	101.51 ± 14.68^b^	228.67 ± 56.47^a^
Lithocholic acid	NBW	309.90 ± 47.20	338.40 ± 38.31	443.74 ± 78.76
	IUGR	334.91 ± 48.28^b^	290.29 ± 37.27^b^	503.53 ± 100.52^a^
α-muricholic acid	NBW	2.29 ± 0.57	1.16 ± 0.30	1.99 ± 0.77
	IUGR	1.84 ± 0.46^a^	1.23 ± 0.27^ab^	0.83 ± 0.25^b^
β-muricholic acid	NBW	0.14 ± 0.03	0.06 ± 0.01	0.08 ± 0.02
	IUGR	0.15 ± 0.04	0.06 ± 0.01	0.09 ± 0.02

Data are presented as means ± SEM (*n* = 8−10). ^a−b^Different superscript letters in the same row showed significantly different (*P* < 0.05) at 25, 50, and 100 kg growth stages (*P* < 0.05); 25, 50, and 100 kg represent the stages when the average BW of NBW pigs reached at 25, 50, and 100 kg BW. *NBW*, normal birth weight. *IUGR*, intrauterine growth retardation

At the growing-finishing stage, in the NBW pigs, the concentrations of glycohyodeoxycholic acid, glycohyocholic acid, isodeoxycholic acid, and β-muricholic acid were decreased (*P* < 0.05), and α-muricholic acid (*P* = 0.09) was tended to decrease at the 50 kg BW stage compared with those at the 25 kg BW stage; the concentrations of dehydrolithocholic acid, isolithocholic acid, isodeoxycholic acid, and β-muricholic acid were increased (*P* < 0.05), while isodeoxycholic acid (*P* < 0.05) and β-muricholic acid (*P* = 0.09) were decreased at the 100 kg BW stage compared with those at the 25 kg BW stage; the concentration of glycodeoxycholic acid was increased (*P* < 0.05), and the concentrations of apocholic acid (*P* = 0.07) and dehydrolithocholic acid (*P* = 0.07) had an increasing tendency at the 100 kg BW stage compared with those at the 50 kg BW stage. In the IUGR pigs, the concentrations of apocholic acid and β-muricholic acid were decreased (*P* < 0.05) at the 50 kg BW stage compared with those at the 25 kg BW stage; the concentration of isolithocholic acid was increased (*P* < 0.05), and taurochenodeoxycholic acid (*P* = 0.09) and α-muricholic acid (*P* = 0.06) were trended to decrease at the 100 kg BW stage compared with those at the 25 kg BW stage; the concentrations of dehydrolithocholic acid, isolithocholic acid (*P* < 0.05), lithocholic acid (*P* = 0.07), taurolithocholic acid (*P* = 0.06), and taurochenodeoxycholic acid (*P* = 0.09) were increased at the 100 kg BW stage compared with those at the 50 kg BW stage.

### Correlation between colonic bile acid derivatives and microbiota at different growth stages

**Fig. 6:**
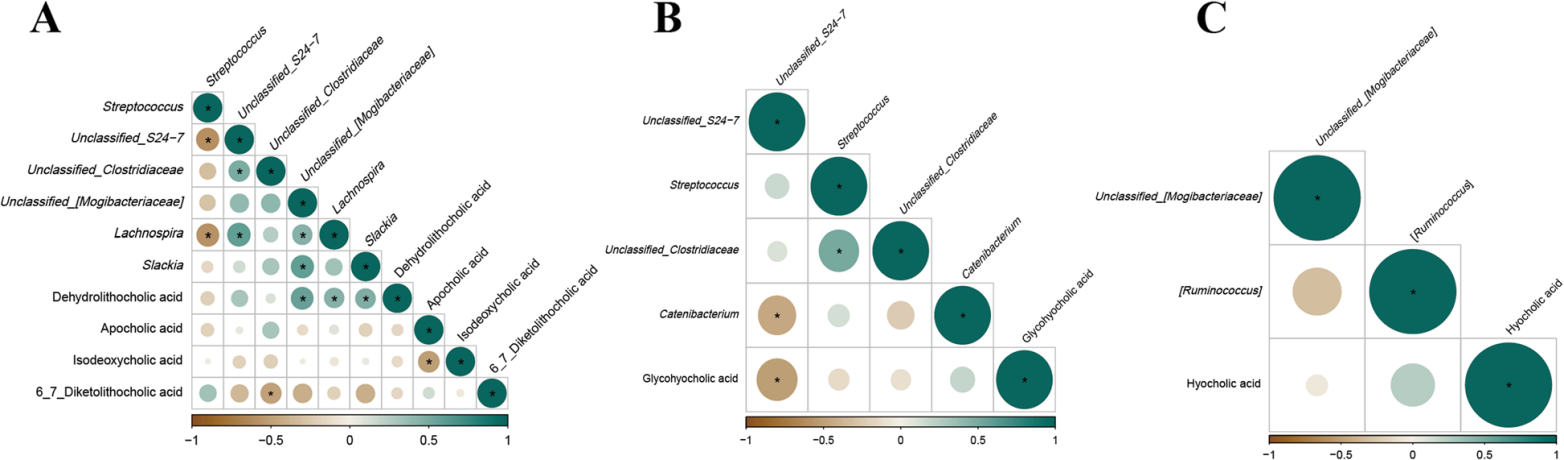
Correlation of bacteria abundances and bile acid derivatives in the colonic contents of intrauterine growth retardation (IUGR) pigs and normal birth weight (NBW) pigs at different growth stages. *P < 0.05. The green color represents a significant positive correlation, and the brown color represents a significant negative correlation. (**A)**, (**B**), and (**C**) represent the relationship between different bacterial abundance and bile acid derivatives in colonic contents at the 25, 50, and 100 kg growth stages

The Spearman’s correlations between different bile acid derivatives and microbiota abundances at the genus level in the colon are shown in Fig. 6. The results showed that *Unclassified_[Mogibacteriaceae], Lachnospira*, and *Slackia* abundances were negatively correlated (*P* < 0.05) with dehydrolithocholic acid, while *Unclassified_Clostridiaceae* abundance was positively correlated (*P* < 0.05) with 6,7-diketolithocholic acid at the 25 kg BW stage. Glycohyocholic acid was positively correlated (*P* < 0.05) with *Unclassified*_*S24-7* abundance at the 50 kg BW stage. There were no significant correlations observed at 7, 21, and 28-day-old and the 100 kg BW stage.

**Fig. 7:**
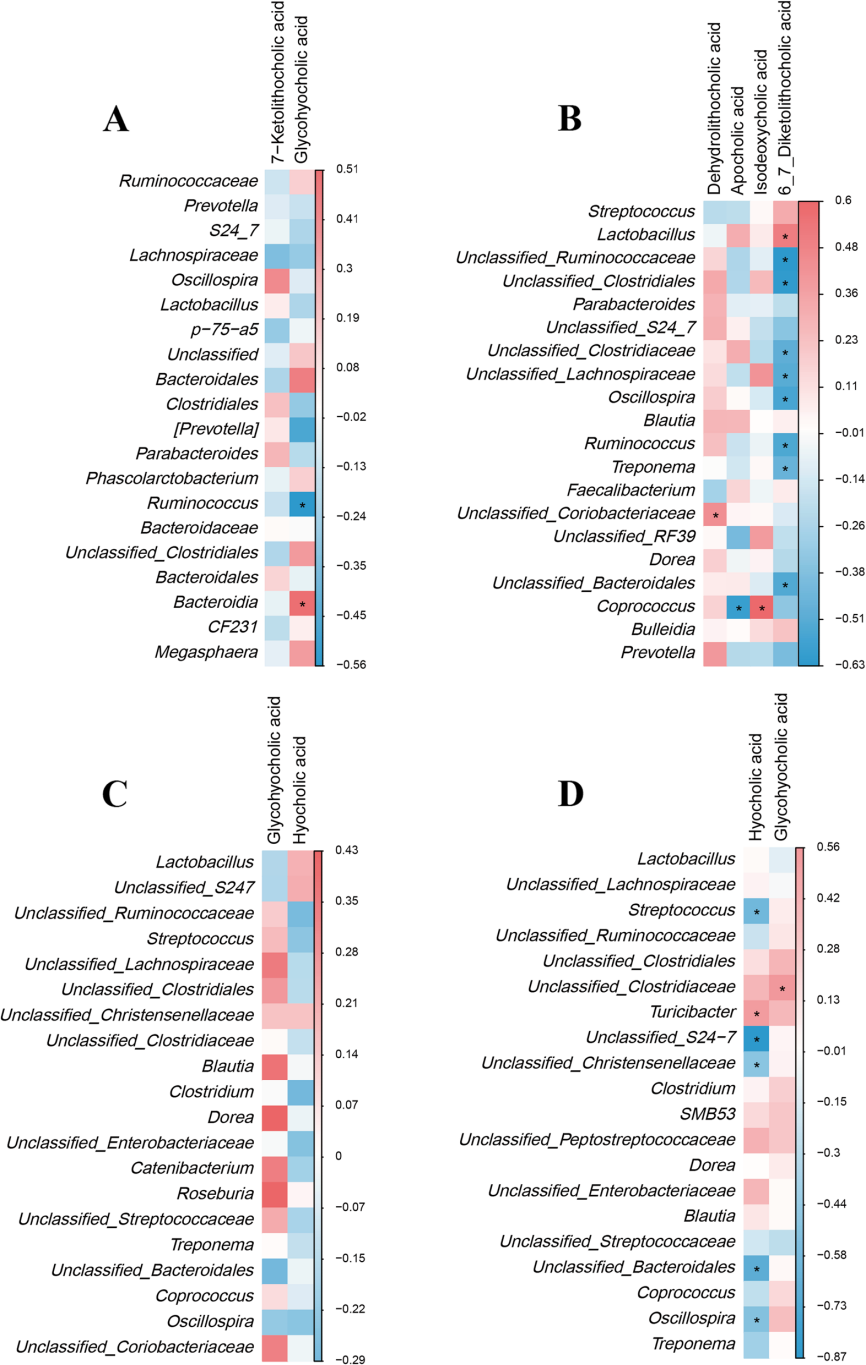
Correlation analysis of the top 20 abundant taxa at the genus level and bile acid derivatives in colon contents of intrauterine growth retardation (IUGR) pigs and normal birth weight (NBW) pigs at different growth stages. *P < 0.05. The red color represents a significantly positive correlation, and blue color represents a significantly negative correlation. (**A)**, (**B**), (**C**), and (**D**) represent the relationship between top 20 bacterial abundances and different bile acid derivatives in colonic contents at 28-day-old, and the 25, 50, and 100 kg growth stages, respectively

In addition, Spearman’s correlation analysis was performed between different bile acid metabolites and the top 20 abundant taxa at the genus level (Fig. 7). The results showed that glycohyocholic acid was positively correlated with *Bacteroidia* abundance while negatively correlated with *Ruminococcus* abundance at 28-day-old (*P* < 0.05). Dehydrolithocholic acid was positively correlated with *Unclassified*_*Coriobacteriaceae* abundance, as well as isodeoxycholic acid with *Coprococcus* abundance, while apocholic acid was negatively correlated with *Coprococcus* abundance (*P* < 0.05). In addition, 6,7-diketolithocholic acid was positively correlated with *Lactobacillus* abundance while negatively correlated with *Unclassified*_*Ruminococcaceae, Unclassified*_*Clostridiales*, *Unclassified*_*Clostridiaceae, Unclassified*_*Lachnospairaceae*, *Oscillospira*, *Ruminococcus*, *Treponema*, and *Unclassified*_*Bacteroidales* abundances at the 25 kg BW stage (*P* < 0.05). Moreover, hyochocholic acid was negatively correlated (*P* < 0.05) with *Streptococcus*, *Unclassified*_*S24_7*, *Unclassified*_*Christensenellaceae*, *Unclassified*_*Bacteroidales*, and *Oscillospira* abundances while positively correlated (*P* < 0.05) with *Turicibacter* abundance. However, no significant correlations were detected at 7 and 21-day-old and the 50 kg BW stage.

### Effects of IUGR on gene expressions related to hepatic bile acid synthesis

**Fig. 8:**
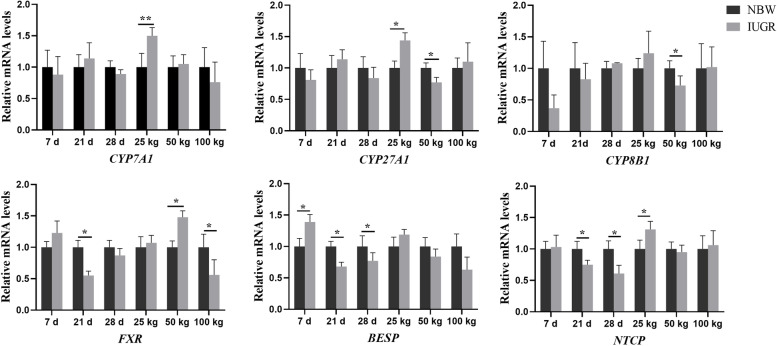
Expression levels of genes related to bile acid synthesis in the liver of intrauterine growth restriction (IUGR) pigs and normal birth weight (NBW) pigs at different growth stages. Data are expressed as means ± SEM (*n* = 8−12). *P < 0.05, **P < 0.01. 7 d, 21 d, and 28 d represent 7, 21, and 28-day-old, respectively; 25 kg, 50 kg, and 100 kg represent the average BW of NBW pigs reached at 25, 50, and 100 kg BW, respectively. *BESP*, bile salt export pump; *CYP7A1*, cytochrome P450 family 7 subfamily A member 1; *CYP8B1*, cytochrome P450 family 8 subfamily B member 1; *CYP27A1*, cytochrome P450 family 27 subfamily A member 1; *FXR*, farnesoid X receptor; *NTCP*, sodium taurocholate cotransporting polypeptide

The effects of IUGR on gene expressions related to hepatic bile acid synthesis are shown in Fig. 8. Compared with the NBW group, the expression levels of *BESP* and *NTCP* were down-regulated (*P* < 0.05) in the IUGR pigs at 21 and 28-day-old, as well as the FXR at 21-day-old. At the 25 kg BW stage, the expression levels of *CYP7A1*, *CYP27A1*, and *NTCP* were up-regulated (*P* < 0.05) in the IUGR pigs compared with the NBW pigs. At the 50 kg BW stage, the expression levels of *CYP27A1* and *CYP8B1* were down-regulated (*P* < 0.05), while the *FXR* was up-regulated (*P* < 0.05) in the IUGR pigs, when compared with the NBW pigs.

### Effects of IUGR on gene expressions related to colonic bile acid receptors

**Fig. 9:**
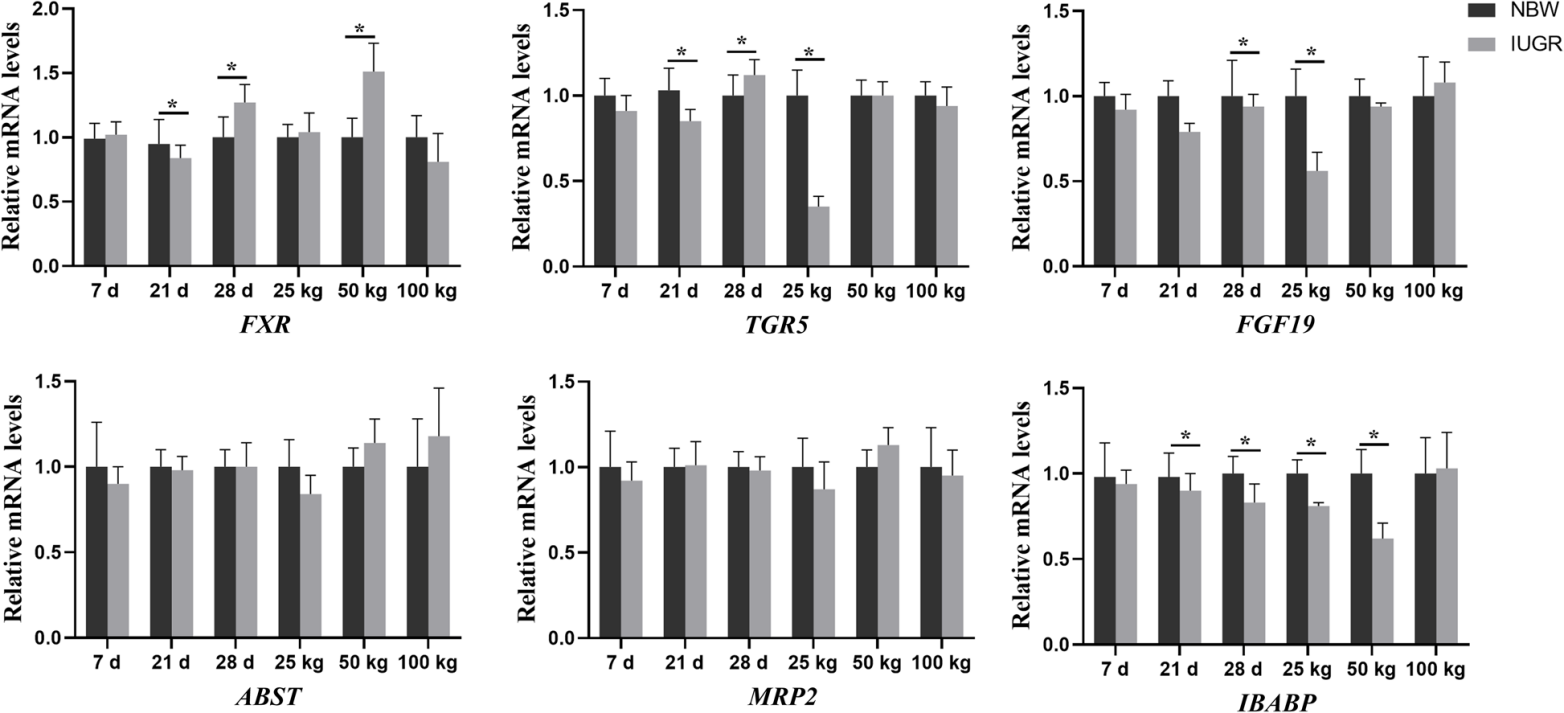
Expression levels of bile acid receptors in the colonic mucosa of intrauterine growth retardation (IUGR) pigs and normal birth weight (NBW) pigs at different growth stages. Data are expressed as means ± SEM (*n* = 8−12). *P < 0.05. 7 d, 21 d, and 28 d represent 7, 21 and 28-day-old; 25 kg, 50 kg, and 100 kg represent the average BW of NBW pigs reached at 25, 50, and 100 kg BW, respectively. *ABST*, apical sodium dependent bile acid transporter; *FXR*, farnesoid X receptor; *FGF19*, fibroblast growth factor 19; *IBABP*, ileal bile acid binding protein; *MRP2*, multidrug resistance protein 2; *TGR5*, G protein-coupled bile acid receptor 1

The effects of IUGR on gene expressions related to colonic bile acid receptors are presented in Fig. 9. Compared with the NBW group, the expression levels of *FXR*, *TGR5*, and *IBABP* were down-regulated (*P* < 0.05) in the IUGR pigs at 21-day-old. At 28-day-old, the expression levels of *FGF19* and *IBABP* were down-regulated while *FXR* and *TGR5* were up-regulated in the IUGR pigs compared with the NBW group (*P* < 0.05). At the 25 kg BW stage, the expression levels of *TGR5* and *FGF19* were down-regulated (*P* < 0.05) in the IUGR pigs compared with the NBW group. At the 50 kg BW stage, the expression level of *IBABP* was down-regulated, while *FXR* was up-regulated in the IUGR pigs compared with the NBW group (*P* < 0.05).

### Effects of IUGR on gene expressions related to hepatic glucose and lipid metabolism

**Fig. 10:**
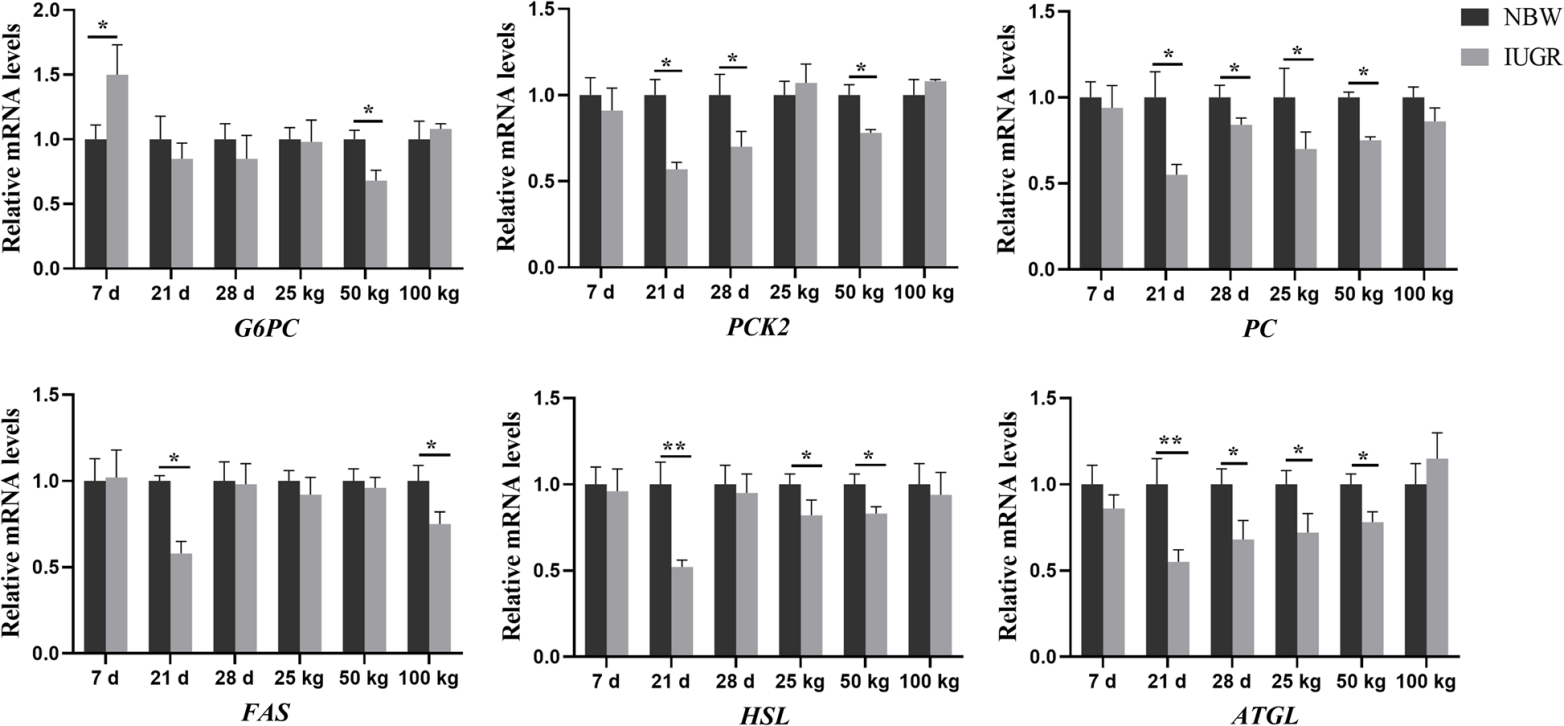
Expression levels of genes related to glucolipid metabolism in the liver of intrauterine growth retardation (IUGR) pigs and normal birth weight (NBW) pigs at different growth stages. Data are expressed as means ± SEM (*n* = 8−12). *P < 0.05, **P < 0.01. 7 d, 21 d, and 28 d represent 7, 21, and 28-day-old; 25 kg, 50 kg, and 100 kg represent the average BW of NBW pigs reached at 25, 50, and 100 kg BW, respectively. *ATGL*, adipose triglyceride lipase; *FAS*, fatty acid synthetase; *G6PC*, glucose-6-Phosphatase; *HSL*, hormone-sensitive lipase; *PC*, pyruvate carboxylase; *PCK2*, phosphoenolpyruvate carboxykinase 2

The effects of IUGR on gene expressions related to hepatic glucolipid metabolism are presented in Fig. 10. Compared with the NBW group, the expression level of *G6PC* was up-regulated (*P* < 0.05) in the IUGR pigs at 7-day-old. The expression levels of *ATGL*, *FAS*, *HSL*, *PC*, and *PCK2* were down-regulated (*P* < 0.05) in the IUGR pigs at 21-day-old, as well as the *ATGL*, *PC*, and *PCK2* at 28-day-old, when compared with the NBW group. At the 25 kg BW stage, the expression levels of *ATGL*, *HSL*, and *PC* were down-regulated (*P* < 0.05) in the IUGR pigs compared with the NBW group. At the 50 kg BW stage, the expression levels of *ATGL*, *G6PC*, *HSL*, *PC*, and *PCK2* were down-regulated (*P* < 0.05) in the IUGR pigs compared with the NBW group. However, there was no significant difference at the 100 kg BW stage (*P* > 0.05).

## Discussion

Newborn IUGR piglets have higher mortality rates and impaired intestinal morphology and function, which reduce the growth performance and increase the risk of infection [19]. The present study indicates that in situation of IUGR, the newborn piglets are characterized in the growing phase by modification of the circulating concentrations of bile acids, expression of genes implicated in bile acid and glucolipid metabolism, and in the bile acid derivatives concentrations in the colon. Furthermore, the measurement of bile acid derivatives in the colonic content of growing pigs, originating from either a situation of IUGR or normal intrauterine growth, is of interest when considering that bile acid-related compounds in feces is associated with the cholesterol status in humans [20].

Total plasma bile acids assay for the evaluation of hepatic function has been available for many years [21], such as abnormal plasma total bile acids indicated impaired hepatic function, bile acid enterohepatic circulation disorder [22], and hepatic inflammation and collagen deposition [23]. The present study showed that plasma total bile acids concentrations were increased at the early stage and decreased in growing-finishing pigs in both NBW and IUGR pigs, especially at 28-day-old and the 25 kg BW stage. Notably, the IUGR pigs had higher plasma total bile acids concentrations than that of the NBW pigs. A previous study showed that IUGR could reduce the weight of the liver and had adverse effects on hepatic mitochondrial function and redox status in the IUGR pigs [24]. These results indicated that abnormal bile acids levels were associated with hepatic dysfunction in IUGR pigs.

The impaired intestinal integrity, immune system dysfunction, and diarrhea are often results in piglets during weaning [25], which can change the intestinal microecological balance and their metabolites, especially for IUGR pigs [26]. Bile acids are one of the important metabolites in the intestine. In the present study, compared with the pigs at 21-day-old, the concentrations of ursodeoxycholic acid, β-muricholic acid, and ursocholic acid were significantly increased between the two groups at 28-day-old. Previous studies showed that ursodeoxycholic acid had protective effects, including enhancing antioxidants [27], protecting against intestinal inflammation, and promoting barrier function [28]. Moreover, the concentrations of cholic acid was significantly decreased in the IUGR pigs compared with the NBW pigs at 21-day-old. The glycohyodeoxycholic acid had a higher concentration in the IUGR pigs than that in the NBW pigs at 28-day-old, which is consistent with the fact that glycohyodeoxycholic acid had the highest proportion in pig’s bile powder and accounted for 18%−22% of the content [29]. These findings implied that the weaning increased the concentration of colonic ursodeoxycholic acid, which can protect the intestinal epithelium. Increased hyodeoxycholic acid may be associated with blood glucose reduction of IUGR pigs at weaning. Further studies are needed to explore the relationship between bile acid metabolism and glucose metabolism in piglets at weaning.

Previous studies showed that higher concentrations of bile acids could result in inflammation and intestinal damage [30]. Furthermore, hyocholic acid and its derivatives play an important role in blood glucose regulation [31]. Hyodeoxycholic acid had the same effects in decreasing blood cholesterol and triglycerides [32]. In the present study, a total of 29 bile acid derivatives were detected in the colon and the proportion of bile acid derivatives were dynamically changed with the changes of growth stages in the two groups. In addition, hyodeoxycholic acid showed a higher concentration at the growing-finishing stage in the NBW and IUGR pigs, which is in accordance with the concentrations of hyocholic acid and its derivatives [31].

Early colonization of intestinal microbiota plays an important role in the development of the intestinal immune system and nutrient absorption of the host [33], especially for IUGR pigs. Our previous study showed that *Ruminococcus* and *Oscillospira* abundances were positively associated with the BW of IUGR piglets [17]. Furthermore, bile acid species can affect and regulate gut microbial species composition [34]. The present study showed that several colonic bile acids were correlated with the colonic microbiota composition, notably glycohyocholic acid. This compound latter showed a negative correlation with the *Ruminococcus* abundance, while positively correlated with the *Bacteroidia* and *Unclassified*_*S24-7* abundances. Moreover, our study showed that the hyocholic acid was negatively correlated with *Streptococcus*, *Unclassified_S24_7*, *Unclassified_Christensenellaceae*, *Unclassified_Bacteroidales*, and *Oscillospira* abundances, while positively correlated with *Turicibacter* abundance. These findings indicate that the changes of colonic bile acid derivatives were influenced by intestinal microbiota and may be associated with the growth performance of pigs. Further studies are needed to explore the effects of bile acid supplementation on intestinal microbiota and growth performance of IUGR pigs.

In mammals, the synthesis of the majority of bile acids involves the classic pathway in which hydroxylation of the cholesterol steroid nucleus is performed by *CYP7A1* [35]. Cholesterol can be converted into primary bile acids in the hepatocyte by a complex biochemical pathway involving a series of hepatic enzymes, such as *CYP7A1*, *CYP8B1*, and *CYP27A1,* which can promote the primary bile acid synthesis [36]. Sodium-taurocholate co-transporting polypeptide (*NTCP*) plays a vital role in transporting bile acids from the sinusoidal blood into the hepatocyte [37]. In the present study, the expression levels of *CYP7A1* and *CYP27A1* were up-regulated in the IUGR pigs at the 25 kg stage, and *NTCP* was down-regulated at 21 and 28-day-old. These results were compatible with the view that IUGR increased hepatic bile acid synthesis and decreased bile acids return to hepatocyte, which would explain the increased plasma total bile acids. Similar findings were also observed in the bile acid metabolism of humans [38]. However, further studies are needed to explore the effects of IUGR on bile acid receptors.

Farnesoid X receptor, a nuclear hormone receptor, can modulate bile acid synthesis in liver by forming a heterodimer with retinoid X receptor (RXR) and binding directly to the regulatory elements of its target genes, as well as by regulating *SHP* [39]. In the present study, the hepatic expression level of *FXR* was up-regulated, while *CYP8B1*and *CYP27A1* were down-regulated in the IUGR pigs at the 50 kg BW stage. This likely indicates that bile acid synthesis was reduced via* FXR* pathway. Further studies are necessary to explore the effect of IUGR on *FXR-SHP* pathway in bile acid metabolism.

Bile acids are reabsorbed into the enterocyte via the apical sodium dependent bile acid transporter (*ASBT*) [40]. The *ASBT* conjugated bile salts into the enterocyte, which interacts with *I-BABP* within the cytosol [41]. Therefore, *FXR* also regulates the release of *FGF-19*, which suppresses bile acid synthesis by repressing *CYP7A1* expression in the hepatocyte [42]. In the present study, the hepatic expression level of *FXR* was up-regulated, while *FGF19* and *IBABP* were down-regulated, which indicated that IUGR may activate intestinal *FXR*, induce expression of *FGF19*, and reduce bile acid reabsorption. Moreover, the expression level of *FXR* was up-regulated, and *FGF19* was down-regulated in the colonic mucosa. This is consistent with the fact that the *FXR* activation indirectly promoted hepatic bile acid synthesis by decreasing the plasma *FGF19* level [43]. These findings indicate that bile acid metabolism was regulated by *FXR* in the intestine.

The IUGR is associated with adverse effects on energy metabolism [44]. Previous studies showed that piglets with IUGR had higher levels of insulin resistance and hepatic lipid accumulation [45]. In the present study, the expression levels of hepatic *HSL* and *ATGL* were down-regulated at 21-day-old and the 25 and 50 kg BW stages. Moreover, the expressions of genes related to gluconeogenesis were down-regulated in the IUGR pigs, including *PC*, *G6PC*, and *PCK2* at 28-day-old and the 50 kg stage. These findings indicate that IUGR could reduce gluconeogenesis and lipid catabolism and increase lipid storage. Furthermore, *FXR* modulates several physiological processes, such as lipid and glucose homeostasis, as well as the inflammatory response [46]. The *TGR5*, as a metabolic regulator, is involved in energy homeostasis, bile acid homeostasis, and glucose metabolism [47]. Moreover, *TGR5* may also induce the secretion of glucagon-like peptide-1 (*GLP-1*), improve liver and pancreatic function, and enhance glucose tolerance [48]. Considering the regulation of bile acid receptors in glycolipid metabolism, the involvement of bile acid receptors might be a potential feature in IUGR pigs. However, further studies are needed to confirm the exact mechanism.

## Conclusion

In summary, IUGR affects bile acid metabolic pathways in pigs and the profile of colonic bile acid derivatives. Bile acid derivatives were associated with changes in intestinal microbiota composition and hepatic bile acid synthesis, together with putative decreased gluconeogenesis and lipid catabolism in pigs. These findings suggested that bile acids could be potential feed additives to improve offspring’s intestinal health and growth rates. In addition, this study also provides a reference for bile acid and energy metabolism of IUGR pigs at different growth stages.

## Supplementary Information

**Additional file1: Table S1.** The composition and nutrient levels of sows' diets (as-fed basis).

**Additional file 2: Table S2.** The composition and nutrient levels of piglets’ creep diets (as-fed basis).

**Additional file 3: Table S3.** The composition and nutrient levels of pigs’ diets (as-fed basis).

## Data Availability

The original contributions presented in the study are included in the article/Supplementary Material, and further inquiries can be directed to the corresponding author.

## Abbreviations

BW: Body weight
IUGR: Intrauterine growth restriction
LEfSe: Linear discriminant analysis effect size
NBW: Normal birth weight
OPLS-DA: Orthogonal projections to latent structures-discriminate analysis
PCA: Principal component analysis
SCFAs: Short-chain fatty acids
