# Supplementary material for: Intrauterine growth retardation affects liver bile acid metabolism in growing pigs: effects associated with the changes of colonic bile acid derivatives
Source: J Anim Sci Biotechnol. 2022 Nov 2;13:117. doi: 10.1186/s40104-022-00772-6 (PMC9628178; doi:10.1186/s40104-022-00772-6)
Supplement: Supplementary file 1 — Additional file1: Table S1. The composition and nutrient levels of sows' diets (as-fed basis). [file 40104_2022_772_MOESM1_ESM.docx]

**Supplementary Table 1** Composition and nutrient levels of sows' diets (as-fed basis)

| Items | Pregnant sows’ diet (%) | Lactating sows’ diet (%) |
| --- | --- | --- |
| **Ingredients** |  |  |
| Corn | 60.30 | 58.65 |
| Wheat bran | 23.50 | 5.00 |
| Soybean meal | 12.00 | 20.50 |
| Soybean oil |  | 4.00 |
| Enzymic protein powder |  | 3.00 |
| Wheat flour |  | 2.00 |
| Imported fish meal |  | 2.50 |
| Lys | 0.12 | 0.15 |
| Val |  | 0.10 |
| Thr | 0.03 | 0.05 |
| Antimildew-agent | 0.05 | 0.05 |
| Pregnant sows’ premix^a^ | 4.00 |  |
| Lactating sows’ premix^b^ |  | 4.00 |
| Total | 100.00 | 100.00 |
| Nutrient levels^c^ | | |
| Digestible energy (MJ/kg) | 15.23 | 15.56 |
| Crude protein | 14.17 | 19.78 |
| Crude fiber | 3.60 | 3.54 |
| Crude ash | 5.61 | 5.95 |
| Threonine | 0.68 | 0.99 |
| Lysine | 0.98 | 1.53 |
| Methionine | 0.12 | 0.16 |

^a^ The pregnant sow’s premix provides the following per kilogram of the diet: vitamin A 10,000 IU, vitamin D 2,500 IU, vitamin E 100 IU, vitamin K 2.0 mg, vitamin B_2_ 10 mg, vitamin B_6_ 1.0 mg, vitamin B_12_ 50 μg, choline chloride 1,500 mg, Fe (FeSO_4_ H_2_O) 80 mg, Cu (CuSO_4_ 5H_2_O) 20 mg, Zn (ZnO) 100 mg, Mn (MnSO_4_ H_2_O) 45 mg, I (KIO_3_) 0.7 mg, Se (Na_2_SeO_3_) 0.25 mg.

^b^ The lactating sow’s premix provides the following per kilogram of the diet: vitamin A 15,000 IU, vitamin D 3,200 IU, vitamin E 50 IU, vitamin K 4.0 mg, vitamin B_1_ 4.0 mg, vitamin B_2_ 10 mg, vitamin B_6_ 3.0 mg, vitamin B_12_ 20 μg, choline chloride 800 mg, Fe (FeSO_4_ H_2_O) 120 mg, Cu (CuSO_4_ 5H_2_O) 20 mg, Zn (ZnO) 112 mg, Mn (MnSO_4_ H_2_O) 24 mg, I (KIO_3_) 0.5 mg, Se (Na_2_SeO_3_) 0.4 mg.

^c^ Nutrient levels were calculated values.
