# Supplementary material for: Intrauterine growth retardation affects liver bile acid metabolism in growing pigs: effects associated with the changes of colonic bile acid derivatives
Source: J Anim Sci Biotechnol. 2022 Nov 2;13:117. doi: 10.1186/s40104-022-00772-6 (PMC9628178; doi:10.1186/s40104-022-00772-6)
Supplement: Supplementary file 2 — Additional file 2: Table S2. The composition and nutrient levels of piglets’ creep diets (as-fed basis). [file 40104_2022_772_MOESM2_ESM.docx]

**Supplementary Table 2** Composition and nutrient levels of piglets’ creep diets (as-fed basis)

| Items | Creep diet (%) |
| --- | --- |
| Extruded corn | 25.00 |
| Whey powder | 20.00 |
| Broken white rice | 15.00 |
| Extruded soybean | 10.00 |
| Fermented soybean meal | 5.00 |
| Fish meal | 5.00 |
| Soybean meal | 3.00 |
| Soybean oil | 2.00 |
| Soybean protein | 2.00 |
| Citric acid | 2.00 |
| Egg powder | 1.00 |
| Premix^a^ | 2.00 |
| Total | 100.00 |
| Nutrient levels^b^ |  |
| Digestible energy（MJ/kg） | 14.49 |
| Crude protein | 18.50 |
| Digestible lysine | 1.40 |
| Digestible methionine | 0.50 |
| Digestible threonine | 0.90 |
| Digestible tryptophan | 0.20 |

^a^ The premix provided the following per kg of diets: vitamin A 8,000 IU, vitamin D_3_ 1,228 IU, vitamin E 15 IU, vitamin K_3_ 3.0 mg, vitamin B_1_ 1.3 mg, vitamin B_2_ 3.1 mg, vitamin B_6_ 1.2 mg, calcium pantothenate 13.4 mg, choline chloride 500 mg, biotin 0.11 mg, niacin 25 mg, folic acid 0.68 mg, VB_12_ 0.03 mg, Fe (FeSO_4_ H_2_O) 120 mg, Cu (CuSO_4_ 5H_2_O) 10 mg, Zn (ZnO) 130 mg, Mn (MnSO_4_ H_2_O) 100 mg, I (KIO_3_) 0.3 mg, Se (Na_2_SeO_3_) 0.3 mg, Lys·HCl (78%) 3 g, Met 2.5 g, L-Thr 6 g, L-Try 1.5 g.

^b^ Nutrient levels were calculated values
