# Supplementary material for: Intrauterine growth retardation affects liver bile acid metabolism in growing pigs: effects associated with the changes of colonic bile acid derivatives
Source: J Anim Sci Biotechnol. 2022 Nov 2;13:117. doi: 10.1186/s40104-022-00772-6 (PMC9628178; doi:10.1186/s40104-022-00772-6)
Supplement: Supplementary file 3 — Additional file 3: Table S3. The composition and nutrient levels of pigs’ diets (as-fed basis). [file 40104_2022_772_MOESM3_ESM.docx]

**Supplementary Table 3** Composition and nutrient levels of pigs’ diets (as-fed basis)

| Items | Nursery pig feed (%)  (28–69 day of age) | Growing pig feed (%)  (70–103 day of age) | Finishing pig feed (%)  (104–165 day of age) |
| --- | --- | --- | --- |
| Ingredients |  |  |  |
| Corn | 60.00 | 61.00 | 61.17 |
| Soybean meal | 27.50 | 25.00 | 25.50 |
| Barley | 6.00 | 8.00 | 8.00 |
| Soybean oil | 2.00 | 1.50 | 1.00 |
| Lysine | 0.16 | 0.18 | 0.13 |
| ~~Dicalcium phosphate~~ | ~~0.10~~ | ~~0.10~~ | ~~0~~ |
| Threonine | 0.10 | 0.07 | 0.08 |
| Methionine | 0.02 | 0.03 | 0.00 |
| Antioxidant | 0.02 | 0.02 | 0.02 |
| Antimildew agent | 0.10 | 0.10 | 0.10 |
| Nursery pigs premix^a^ | 4.00 | 0 | 0 |
| Growing-finishing pig premix^b^ | 0 | 4.00 | 4.00 |
| Total | 100.00 | 100.00 | 100.00 |
| Nutrient levels^c^ |  |  |  |
| Digestible energy, MJ/kg | 13.91 | 13.77 | 13.64 |
| Crude protein | 17.20 | 16.40 | 16.50 |
| Crude fat | 4.70 | 4.30 | 3.80 |
| Crude fiber | 2.70 | 2.70 | 2.80 |
| Digestible lysine | 1.17 | 1.08 | 1.05 |
| Digestible methionine | 0.33 | 0.30 | 0.28 |
| Digestible threonine | 0.77 | 0.71 | 0.73 |
| Total calcium | 0.77 | 0.74 | 0.66 |
| Total phosphorus | 0.56 | 0.52 | 0.45 |

^a^ Nursery pig premix supplied per kg feed: vitamin A 8,000 IU, vitamin D_3_ 228 IU, vitamin E 15 IU, vitamin K_3_ 3.0 mg, vitamin B_1_ 1.3 mg, vitamin B_2_ 3.1 mg, vitamin B_6_ 1.2 mg, vitamin B_12_ 0.03 mg, calcium pantothenate 13.4 mg, choline chloride 500 mg, Fe (FeSO_4_ H_2_O) 120 mg, Cu (CuSO_4_ 5H_2_O) 10 mg, Zn (ZnO) 130 mg, Mn (MnSO_4_ H_2_O) 100 mg, I (KIO_3_) 0.3 mg, and Se (Na_2_SeO_3_) 0.3 mg.

^b^ Growing-finishing pig premix supplied per kg feed: vitamin A 15,000 IU, vitamin D_3_ 200 IU, vitamin E 50 IU, vitamin K_3_ 4.0 mg, vitamin B_1_ 4.0 mg, vitamin B_2_ 10 mg, vitamin B_6_ 3.0 mg, vitamin B_12_ 0.04 mg, calcium pantothenate 20.0 mg, choline chloride 800 mg, Fe (FeSO_4_ H_2_O) 120 mg, Cu (CuSO_4_ 5H_2_O) 20 mg, Zn (ZnO) 112 mg, Mn (MnSO_4_ H_2_O) 124 mg, I (KIO_3_) 0.5 mg, Se (Na_2_SeO_3_) 0.4 mg.

^c^ Nutrient levels were calculated values
